# Traditional masculinity and aggressive behavior: the influence of gender norms and educational attainment

**DOI:** 10.3389/fpsyg.2025.1638171

**Published:** 2026-01-05

**Authors:** Mats Najström, Remi Chamoun, Karin Lindqvist

**Affiliations:** Department of Psychology, Stockholm University, Stockholm, Sweden

**Keywords:** aggression, dominance, education, gender norms, masculinity, toughness

## Abstract

**Introduction:**

Traditional masculinity norms have been consistently linked to aggression in men, yet relatively few studies have examined how specific masculinity dimensions relate to distinct forms of aggression within structural and social contexts. Drawing on hegemonic masculinity and gender role strain theory, this study conceptualized masculinity as a socially constructed and contextually reinforced set of ideals influencing emotional and behavioral regulation. The aim was to examine how distinct masculinity dimensions relate to multiple aggression domains, while considering educational attainment and geographic setting as contextual factors.

**Methods:**

A sample of 229 Swedish men aged 18 to 40 completed the Male Role Norms Inventory–Short Form (MRNI-SF) and the Swedish Universities Scales of Personality (SSP), assessing seven masculinity dimensions and five aggression domains: mistrust, irritability, verbal trait aggression, physical trait aggression, and social desirability. A multivariate general linear model (GLM) was conducted, followed by univariate analyses.

**Results:**

The multivariate GLM revealed significant multivariate effects for the masculinity dimensions Restrictive Emotionality, Dominance, and Toughness, as well as for Educational Attainment. Univariate analyses showed that Restrictive Emotionality and Dominance predicted higher levels of Mistrust, whereas Dominance and Toughness were associated with higher Physical Trait Aggression. Avoidance of Femininity showed a positive unadjusted association with Verbal Trait Aggression, although this effect did not remain significant after correction. Educational Attainment demonstrated consistent main effects, with lower education associated with higher scores on Mistrust and Physical Trait Aggression. Geographic Setting did not show significant effects.

**Discussion:**

These findings indicate that aggression is shaped by a combination of psychological dispositions and socially reinforced gender expectations, with different masculinity dimensions uniquely associated with specific forms of aggression. The results underscore the importance of structural context, particularly educational environments, in shaping the internalization and behavioral expression of masculine norms. Interventions that promote emotional competence and critical engagement with gender roles, especially within educational settings, may help reduce male aggression and support healthier expressions of masculinity. Future research should adopt intersectional and longitudinal approaches to further clarify how gender, class, and cultural background interact to shape masculinity and its behavioral outcomes.

## Introduction

Aggressive behavior does not arise in isolation; it often reflects broader social and cultural patterns. Traditional masculinity ideals which emphasize control, emotional restriction, and physical dominance, are linked to higher levels of aggression in men (Logoz et al., 2023). These ideals are not evenly distributed across society; research suggests they are more prevalent among men with lower educational attainment and those living in rural or less urbanized areas (Silva, 2022). Such environments may reinforce narrow definitions of masculinity, increasing the social acceptance of aggression as a legitimate male behavior. Over time, these masculinity norms may not only predict individual aggression but also foster social climates where aggression is normalized and passed on to younger generations. Studies have shown that children exposed to aggressive parental behavior or rigid gender roles may internalize these models through social learning, perpetuating a cycle of interpersonal violence (Bandura, 1977; Perales et al., 2021).

Masculinity is widely understood as a culturally embedded construct that encompasses societal norms and expectations about how boys and men should behave, think, and feel (Levant and Richmond, 2008). Traditional masculinity emphasizes traits such as emotional restriction, dominance, physical toughness, and heteronormativity. These ideals often marginalize behaviors associated with vulnerability, emotional openness, and non-dominant forms of self-expression.

Connell’s (2005) influential concept of hegemonic masculinity conceptualizes masculinity as a culturally idealized ideal grounded in dominance, hierarchy, and the rejection of traits associated with femininity. Although not necessarily the most common form of masculinity enacted in everyday life, it is the most socially validated and institutionally reinforced expression of manhood, as it is upheld and perpetuated through key societal structures such as media, education, and the workplace (Connell and Messerschmidt, 2005). This hegemonic model prioritizes control, competitiveness, and emotional restraint, traits that are frequently associated with the normalization of aggression and the marginalization of alternative masculinities. Research by Silva (2022) suggests that adherence to these ideals is particularly pronounced among men with lower levels of educational attainment, indicating that class-based cultural norms play a significant role in shaping how masculinity is constructed and performed. Supporting this view, a cross-national study by Ola (2018) found that lower educational status among men was strongly associated with increased domestic violence against female partners, particularly when combined with alcohol use. These findings underscore the role of educational disadvantage not only in reinforcing hegemonic masculine ideals but also in facilitating their expression through aggressive behavior. Together, these studies suggest that limited access to critical gender discourse and emotional regulation resources may intensify the internalization of rigid masculine norms. This dynamic is often linked to lower educational attainment and may increase the likelihood of aggression, particularly in contexts where such behavior is culturally tolerated or overlooked.

Building on a socio-cultural foundation, recent research has further illuminated how dominant masculinity norms contribute to emotional dysregulation and aggressive behavior. Malonda et al. (2023) emphasize that core masculine ideals like control, competitiveness, and emotional stoicism are consistently linked to violence and aggression. These findings align with Pleck’s (1995) Gender Role Strain Paradigm (GRSP), which identifies three types of strain that arise from attempting to conform to masculine ideals: discrepancy strain, dysfunction strain, and trauma strain. Each form contributes to emotional suppression and, ultimately, heightened aggression (Levant and Richmond, 2016). Supporting this framework, Gerdes and Levant (2017) identify masculine gender role stress as a reliable predictor of violence, underscoring the psychological costs of rigid gender norms.

The cumulative impact of social and cultural mechanisms that reinforce traditional masculinity can be detrimental to emotional development. A recent study by Sikweyiya et al. (2025) found that early masculine socialization inhibits emotional expression and discourages help-seeking, thereby contributing to psychological strain and aggression. These findings reinforce GRSP’s claim that perceived failure to meet masculine expectations results in distress, which may lead to aggression as a compensatory response to emotional discomfort, inadequacy, or status threat. This interpretation is also supported by Vandello and Bosson (2013), who conceptualize masculinity as a precarious identity that must be continually demonstrated, often through dominance and risk-taking.

Further empirical support for this conceptualization spans several decades. Eisler and Skidmore (1987) found that men endorsing traditional masculine norms exhibited significantly greater hostility and interpersonal aggression. Similarly, Malonda-Vidal et al. (2021) reported that adherence to such norms among adolescents predicted both reactive and proactive aggression, a pattern mediated by emotional regulation deficits and reduced self-efficacy. Logoz et al. (2023) extend this line of inquiry by showing that stronger adherence to traditional masculine norms is linked to emotional suppression, lower self-compassion, alexithymia, and increased risk of physical domestic violence.

While internalized norms play a central role, masculinity is also actively constructed through social interaction and institutional reinforcement. Paechter (2007) and Pettersson (2020) emphasize that masculinity is performed and affirmed within specific cultural and interpersonal contexts. In particular, male peer groups and educational institutions play key roles in reinforcing traits such as dominance, aggression, and emotional restraint (Kehily, 2001; Frosh et al., 2017). These settings frame aggression as a valid form of masculine expression.

Broader frameworks have also emphasized the fluid and context-dependent nature of masculinity. Wong and Wang (2022) propose a multidimensional model with five interrelated domains: self-ascribed, other-ascribed, situational, performative, and macro masculinities. This model highlights that masculinity is not monolithic but is shaped by individual interpretation, social interaction, and institutional and geographic context. Supporting this, Silva (2022) found that men in rural areas tend to endorse more traditional masculinity norms than their urban counterparts, illustrating the influence of socio-spatial setting.

Masculine identity is further sustained through narrative. Pettersson (2020) shows how autobiographical storytelling selectively reinforces hegemonic ideals by emphasizing strength and control while omitting vulnerability. These narratives are underpinned by culturally internalized stereotypes that shape how men view themselves and others. This interplay between personal identity and cultural messaging helps explain why even men who do not fully endorse traditional masculinity may still conform to its scripts in social settings.

Finally, men often navigate a split between positive traits (e.g., autonomy, leadership) and negative ones (e.g., aggression, dominance). Steinberg and Diekman (2016) and Hentschel et al. (2019) found that men frequently align with agentic characteristics such as assertiveness and competitiveness, reinforcing conformity to traditional masculine roles. Collectively, these mechanisms reveal how masculinity operates as a culturally embedded and structurally reinforced system that shapes both emotional development and the enactment of aggression.

Taken together, research suggests that masculinity is a complex, socially constructed phenomenon, with traditional norms consistently linked to emotional suppression and aggression. Yet there remains a need to clarify the mechanisms through which masculinity norms translate into aggressive behavior, especially when considering the effects of educational level and geographic context.

Aggression is commonly defined as intentional behavior aimed at harming another individual who wishes to avoid such harm (Allen and Anderson, 2017). This definition encompasses a wide range of verbal, physical, and emotional expressions, and has informed much of the contemporary research in social psychology. It also includes internal processes such as affect (anger, irritability) and cognition (hostile beliefs and expectations) (Santos et al., 2019). For example, the Swedish Universities Scales of Personality (SSP) conceptualizes aggression as a multifaceted construct comprising five interrelated dimensions: social desirability, irritability, mistrust, verbal aggression, and physical aggression.

Grounded in the literature on traditional masculinity and its psychological and behavioral consequences, the present study empirically examines how masculinity norms predict aggression in men. The theoretical framework draws on hegemonic masculinity (Connell and Messerschmidt, 2005), gender role strain (Pleck, 1995), and perspectives on masculinity as a social product (Pettersson, 2020; Paechter, 2007). These frameworks suggest that rigid masculine norms may increase aggression, and that such tendencies are further shaped by broader social and structural contexts, including educational and geographic factors. Although prior research has linked traditional masculinity norms to aggression, limited attention has been given to how these associations manifest across social and structural contexts, particularly with respect to education and geographic setting. The present study extends this work by examining how contextual factors such as educational attainment and geographic location relate to aggression outcomes alongside distinct dimensions of masculinity. Moreover, while several studies have explored general associations between masculinity and aggression, fewer have used validated instruments such as the Male Role Norms Inventory–Short Form (MRNI-SF) and the Swedish Universities Scales of Personality (SSP) to investigate how distinct masculinity dimensions relate to different forms of aggression. By integrating these instruments within a cross-sectional survey framework, the present study empirically examines how traditional masculinity norms and contextual factors such as educational attainment and geographic setting relate to different forms of aggression.

Research Question: To what extent do traditional masculinity norms predict aggressive behavior, and how are these associations related to educational attainment and geographic context?

By addressing this question, the study aims to contribute a more integrated understanding of the individual, cultural, and structural dimensions of masculinity and their role in shaping aggressive behavior. This approach may also offer insights into how rigid masculine ideals are maintained or challenged within different educational and geographic environments.

## Method

### Participants

A total of 530 individuals completed the survey. After applying inclusion criteria which required participants to be male and between 18 and 40 years old, the final sample consisted of 229 men (M = 26.5 years, SD = 4.96). Women, individuals identifying as other genders, and men over 40 were excluded to maintain consistency with the targeted demographic group of the present study. The attrition between the initial respondent pool and the final sample was due to the application of inclusion criteria *post hoc*. These criteria were not explicitly stated in the survey to prevent individuals outside the target demographic (e.g., women or older men) from misreporting demographic information. This approach preserved data integrity and ensured that the final sample accurately represented men aged 18–40. Participants who did not meet these criteria were excluded during data cleaning, resulting in the observed reduction in sample size. Additionally, no item-level missing data occurred, as the survey platform required all items to be completed before submission.

### Ethical considerations

Ethical review and approval was not required for this study on human participants in accordance with local legislation and institutional requirements. This study was carried out in accordance with the Declaration of Helsinki (World Medical Association, 2024) and with national rules and recommendations regarding good research practice. Participation was voluntary and anonymous, and no personal identifiers or sensitive information were collected. Participants received detailed information about the study’s purpose and provided informed consent by selecting a checkbox before proceeding with the survey. Written informed consent was therefore not required in accordance with national legislation and institutional requirements.

### Instruments

Collected demographic variables included gender, age, educational attainment, and geographic location. Educational attainment was measured on a 4-point scale ranging from 1 (elementary school) to 4 (university). Geographic location was also categorized on a 4-point scale based on population size: 1 (large city), 2 (medium sized city), 3 (small sized town), and 4 (rural area).

The Male Role Norms Inventory–Short Form (MRNI-SF) (Levant et al., 2013) includes 21 items across seven domains: Restrictive Emotionality (RE), Self-Reliance through Mechanical Skills (SR), Negativity toward Sexual Minorities (NSM), Avoidance of Femininity (AF), Importance of Sex (IS), Dominance (D), and Toughness (T). Items were rated on a 7-point Likert scale ranging from 1 (strongly disagree) to 7 (strongly agree). The instrument was translated and culturally adapted into Swedish using a standard translation and back-translation process performed by bilingual psychologist’s familiar with gender-norm research. Internal consistency for the seven three-item subscales was acceptable (Cronbach’s *α* = 0.78–0.84). Although a confirmatory factor analysis of the Swedish version was not conducted, subscale interpretations followed the established seven-factor model reported by Levant et al. (2013). This adaptation and the associated reliability indices suggest that the Swedish MRNI-SF provides an adequately reliable measure of traditional masculinity norms for the present analyses.

The Swedish Universities Scales of Personality (SSP) (Gustavsson et al., 2000) was used to assess aggression across five domains: Trait Irritability (TI), Mistrust (M), Verbal Trait Aggression (VTA), Physical Trait Aggression (PhTA), and Social Desirability (SD). Each domain contained seven items rated on a 4-point Likert scale. Although SD is sometimes conceptualized as a response-style indicator, within the SSP it represents a personality trait reflecting prosocial and conforming tendencies, loading negatively on the aggressiveness factor (Gustavsson et al., 2000). In this study, SD was therefore included as an outcome indexing socially regulated aspects of aggression expression.

### Procedure

Data were collected via a Google Forms survey. The questionnaire began with an informed consent statement, followed by demographic questions, the MRNI-SF, and the SSP. The survey was disseminated through social media and direct communication with students and staff at Stockholm University, Stockholm Sweden.

### Statistical analyses

All statistical analyses were conducted using IBM SPSS Statistics (version 29). Preliminary data screening confirmed the absence of missing data or data-entry errors. Descriptive statistics were computed for all study variables, and bivariate correlations were examined to explore preliminary associations among masculinity dimensions and aggression outcomes. Age showed no meaningful associations with any masculinity dimension or aggression outcome (all correlations nonsignificant, *r* < 0.12). Given the restricted age range of the sample (18–40 years), age was therefore excluded from further analyses. Educational attainment and geographic location were included as contextual fixed factors based on their established theoretical and empirical relevance to gender-norm endorsement and aggressive behavior (Silva, 2022; Ola, 2018). These variables were modeled as main effects to capture structural influences on aggression outcomes. Although potential interaction terms between masculinity dimensions and contextual variables were initially explored, they were not retained in the final model due to lack of statistical significance and limited theoretical support for higher-order interactions.

To address the primary research question a multivariate general linear model (GLM) was employed. The analysis included five aggression outcomes (SD, TI, M, VTA, and PhTA) as dependent variables. Seven continuous masculinity dimensions (RE, SR, NSM, AF, IS, D, and T) were entered as covariates, whereas educational attainment and geographic location were included as fixed factors. Multivariate significance was evaluated using Pillai’s Trace, which is generally considered the most robust statistic against violations of normality and heterogeneity of covariance matrices (Tabachnick and Fidell, 2019). For multiplicity control, the Holm–Bonferroni procedure was applied across the five aggression outcomes as a single family of tests for the masculinity predictors, whereas Bonferroni corrections were applied separately to *post hoc* group comparisons for the categorical factors Education and Geography. Because SD is sometimes treated as a response-style indicator, supplementary analyses including SD as a covariate were conducted and showed that the associations between masculinity dimensions and aggression outcomes remained substantively unchanged. Significant multivariate effects were followed by univariate tests of between-subjects effects for each dependent variable. Effect sizes were reported as partial eta squared (η^2^p) for multivariate and univariate effects, and standardized coefficients (*β*) with 95% confidence intervals were obtained from supplementary regressions to provide estimates of magnitude and precision. Model fit was summarized using coefficients of determination (*R*^2^) for each dependent variable.

### Assumptions checks and diagnostics

Prior to analysis, model assumptions were evaluated to ensure robustness of the multivariate GLM. Multivariate normality was assessed using Mahalanobis distances across the five dependent variables (SD, TI, M, VTA, PhTA). All cases fell below the critical *χ*^2^(5) = 20.52 threshold (*p* < 0.001), indicating that the assumption of multivariate normality was adequately met. Linearity between continuous predictors and aggression outcomes was examined through scatterplots of standardized residuals versus predicted values, which showed no systematic deviations. Homogeneity of variance–covariance matrices was verified using Box’s M test, M = 122.64, *F*(105, 3,256) = 0.91, *p* = 0.74, indicating no violation of this assumption. Levene’s tests of equality of error variances were nonsignificant for all dependent variables (*p*s > 0.25), indicating homoscedasticity. Normality of residuals was supported by standardized residual histograms and Q–Q plots. Collinearity diagnostics (Tolerance = 0.34–0.98, VIF = 1.02–2.90) confirmed absence of multicollinearity. Influence statistics (standardized residuals, Cook’s distance, and leverage) were examined in supplementary regressions; no cases exceeded conventional thresholds (no standardized residuals > 3.0, Cook’s *D* > 1.0, leverage > 0.20), indicating no influential or outlying cases (Tabachnick and Fidell, 2019).

## Results

Table 1 summarizes the descriptive statistics for the demographic variables, including educational level and geographic location.

**Table 1:** Participant distribution by educational attainment and geographic location.

Variable	Category	*N*	%
Educational level	(1) Elementary school	25	10.9
	(2) Upper secondary	65	28.4
	(3) Post-sec. Edu.	26	11.4
	(4) University degree	113	49.3
Geographic location	(1) Large city	162	70.7
	(2) Medium-sized city	33	14.4
	(3) Small town	20	8.7
	(1) Rural area	14	6.1

Percentages are based on the total sample size (*N* = 229).

Table 2 presents the bivariate correlations among all study variables. RE, D, and T were moderately interrelated and showed the strongest positive associations with M and PhTA aggression, whereas correlations with SD were small and negative.

**Table 2:** Correlations between masculinity dimensions and aggression outcomes.

Variable	1	2	3	4	5	6	7	8	9	10	11	12
1. RE	—											
2. SR	0.54**	—										
3. NSM	0.32**	0.36**	—									
4. AF	0.56**	0.52**	0.55**	—								
5. IS	0.46**	0.35**	0.46**	0.56**	—							
6. D	0.37**	0.37**	0.69**	0.65**	0.55**	—						
7. T	0.63**	0.63**	0.51**	0.69**	0.43**	0.60**	—					
8. SD	0.05	0.08	0.10	0.03	0.07	0.02	0.05	—				
9. TI	0.14*	0.10	0.12	0.20**	0.17*	0.18*	0.14*	−0.35**	—			
10. M	0.36**	0.20**	0.11	0.26**	0.18**	0.29**	0.27**	−0.28**	0.49**	—		
11. VTA	0.20**	0.13	0.07	0.26**	0.12	0.18**	0.21**	−0.36**	0.52**	0.45**	—	
12. PhTA	0.40**	0.35**	0.25**	0.44**	0.21**	0.42**	0.51**	−0.18**	0.41**	0.45**	0.43**	—

RE, Restrictive emotionality; SR, Self-Reliance through Mechanical Skills; NSM, Negativity toward Sexual Minorities; AF, Avoidance of Femininity; IS, Importance of Sex; D, Dominance; T, Toughness; TI, Trait Irritability; M, Mistrust; VTA, Verbal Trait Aggression; PhTA, Physical Trait Aggression. All correlations are Pearson’s *r*, two-tailed. *p < 0.05, **p < 0.01, *N* = 229.

### Multivariate analysis

A multivariate general linear model (GLM) was conducted to examine the effects of seven masculinity dimensions: RE, SR, NSM, AF, IS, D, T along with, educational attainment, and geographic context on five aggression outcomes: SD, TI, M, VTA, and PhTA. The final model included only main effects, as exploratory interaction terms were nonsignificant and lacked theoretical support for inclusion.

Significant multivariate effects were observed for the following predictors:

- RE (*Pillai’s Trace* = 0.066, *F*(5, 211) = 2.99, *p* = 0.012, η^2^_p_ = 0.07)
- D (*Pillai’s Trace* = 0.077, *F*(5, 211) = 3.51, *p* = 0.005, η^2^_p_ = 0.08),
- T (*Pillai’s Trace* = 0.055, *F*(5, 211) = 2.47, *p* = 0.033, η^2^_p_ = 0.06)
- Education (*Pillai’s Trace* = 0.145, *F*(15, 639) = 2.17, *p* = 0.006, η^2^_p_ = 0.05)

No other predictors reached multivariate significance (see Table 3). Although no significant main effect of geographic location emerged, this result should be interpreted cautiously given the uneven group distribution (70.7% large city, 14.4% medium-sized city, 8.7% small town, 6.1% rural area). A *post hoc* sensitivity analysis (*N* = 229; four groups) showed 80% power to detect effects of at least *f* = 0.22 (η^2^ ≈ 0.046), suggesting adequate power for medium effects but limited sensitivity to smaller differences.

**Table 3:** Multivariate effects of masculinity dimensions, education, and geography on aggression.

Outcomes	Multivariate Test	*F*(df₁, df₂)	*p*	Partial η^2^
**Restrictive emotionality**	**0.066**	**2.997 (5, 211)**	**0.012**	**0.066**
Self-reliance through mechanical skills	0.004	0.151 (5, 211)	0.980	0.004
Negativity toward sexual minorities	0.037	1.634 (5, 211)	0.152	0.037
Avoidance of femininity	0.031	1.364 (5, 211)	0.239	0.031
Importance of sex	0.043	1.905 (5, 211)	0.095	0.043
**Dominance**	**0.077**	**3.507 (5, 211)**	**0.005**	**0.077**
**Toughness**	**0.055**	**2.474 (5, 211)**	**0.033**	**0.055**
**Education**	**0.145**	**2.167 (15, 639)**	**0.006**	**0.048**
Geography	0.061	0.887 (15, 639)	0.579	0.020

Multivariate significance was evaluated using Pillai’s Trace.

Partial η^2^ represents effect size for each omnibus test. Statistically significant results are bolded.

### Univariate effects

Follow-up univariate tests were conducted for each dependent variable to examine the predictors contributing to the multivariate effects. Standardized coefficients (*β*) and 95% confidence intervals for the univariate follow-up analyses are presented in Table 4. These values are reported prior to multiple-comparison adjustments and serve to illustrate the direction and magnitude of effects.

**Table 4:** Associations between masculinity dimensions, education, geography, and aggression outcomes.

Dependent Variable	Significant Predictor(s)	Standardized *β*	95% CI
			[Lower, Upper]
Mistrust	Restrictive emotionality	0.30	[0.14, 0.46]
	Dominance	0.33	[0.12, 0.53]
	Negativity toward sexual minorities	−0.19	[−0.35, −0.02]
	Education	−0.19	[−0.36, −0.04]
Trait irritability	—	—	—
Verbal trait aggression	Avoidance of femininity	0.17	[−0.02, 0.35]
Physical trait aggression	Toughness	0.33	[0.14, 0.49]
	Dominance	0.21	[0.06, 0.38]
	Importance of sex	0.20	[0.04, 0.36]
	Education	−0.19	[−0.37, −0.05]
Social desirability	—	—	—

Standardized coefficients (*β*) and 95% confidence intervals (CIs) are presented prior to Holm–Bonferroni and Bonferroni adjustments.

Mistrust—The model was statistically significant, *F*(13, 215) = 4.86, *p* < 0.001, η^2^_p_ = 0.23.

Significant predictors included:

- Restrictive Emotionality: *F*(1, 215) = 12.04, *p* < 0.001, η^2^_p_ = 0.05
- Dominance: *F*(1, 215) = 10.36, *p* = 0.001, η^2^_p_ = 0.05
- Negativity toward Sexual Minorities: *F*(1, 215) = 4.70, *p* = 0.031, η^2^_p_ = 0.02
- Education: *F*(3, 215) = 2.87, *p* = 0.038, η^2^_p_ = 0.04

Following Holm–Bonferroni adjustment, Restrictive Emotionality and Dominance remained significant predictors of Mistrust. Education also showed a significant main effect after Bonferroni-adjusted *post hoc* tests, with university-educated participants reporting lower levels of mistrust (*p* = 0.022) compared with those with only basic schooling.

Verbal Trait Aggression—The model was significant, *F*(13, 215) = 2.14, *p* = 0.013, η^2^_p_ = 0.11. The only significant predictor was:

- Avoidance of Femininity: *F*(1, 215) = 5.08, *p* = 0.025, η^2^_p_ = 0.02

After Holm–Bonferroni correction, the effect of AF did not remain a statistically significant predictor.

Physical Trait Aggression—This model yielded the strongest effects, F(13, 215) = 8.91, *p* < 0.001, η^2^_p_ = 0.350. Significant predictors included:

- Dominance: *F*(1, 215) = 9.48, *p* = 0.002, η^2^_p_ = 0.042
- Toughness: *F*(1, 215) = 10.55, *p* = 0.001, η^2^_p_ = 0.047
- Importance of Sex: *F*(1, 215) = 6.13, *p* = 0.014, η^2^_p_ = 0.028
- Education: *F*(3, 215) = 3.15, *p* = 0.026, η^2^_p_ = 0.042

Once Holm–Bonferroni adjustments were applied, only Dominance and Toughness remained significant predictors of Physical Trait Aggression, while Education also remained significant following Bonferroni-adjusted *post hoc* tests, with university-educated participants showing lower levels of physical aggression compared with those with only basic schooling (*p* = 0.017, Bonferroni-adjusted). A summary of Holm–Bonferroni adjusted, and Bonferroni-corrected results is presented in Table 5.

**Table 5:** Significant and near-significant univariate results with Holm–Bonferroni and Bonferroni adjustments of aggression outcomes.

Dependent Variable	Predictor	Raw *p*	Adjusted *p*	Adjustment	Interpretation
Mistrust	Restrictive emotionality	< 0.001	**0.005**	Holm	Higher RE → greater M
	Dominance	0.001	**0.005**	Holm	Higher D → greater M
	Negativity toward sexual minorities	0.031	0.155	Holm	Trend (n.s. after Holm)
Physical trait aggression	Dominance	0.002	**0.010**	Holm	Higher D → greater PhTA
	Toughness	0.001	**0.005**	Holm	Higher T → greater PhTA
	Importance of sex	0.014	0.070	Holm	Trend (n.s. after Holm)
Verbal trait aggression	Avoidance of femininity	0.025	0.125	Holm	Trend (n.s. after Holm)
Education (groups)	Education	0.026	**0.017**	Bonferroni	University < basic education lower PhTA; lower M
Geography (groups)	Geography	> 0.10	—	Bonferroni	No significant differences

Holm–Bonferroni were applied to the familywise error rate across aggression outcomes.

Standard Bonferroni adjustments were applied to comparisons involving Education and Geography.

Statistically significant *p*-values after correction for multiple testing bolded.

In sum, Restrictive Emotionality and Dominance remained significant predictors of Mistrust, whereas Dominance and Toughness predicted Physical Trait Aggression. Educational attainment also remained significant after Bonferroni correction, with participants holding university degrees showing lower aggression levels than those with only basic education. No other effects survived correction for multiple comparisons. Collectively, the results indicate that Dominance, Toughness, and Restrictive Emotionality were the most consistent predictors of Mistrust and Physical Trait Aggression, while higher education mitigated these tendencies. Although geographic location was nonsignificant, post hoc sensitivity analyses suggested sufficient power to detect medium effects, implying that smaller contextual influences may have gone undetected. Overall, the findings emphasize how individual adherence to masculine norms and structural factors such as education jointly shape aggressive behavior.

## Discussion

The present study examined how distinct dimensions of traditional masculinity, as well as educational attainment and geographic setting, relate to multiple forms of aggression. The findings lend strong empirical support to theoretical models of hegemonic masculinity (Connell and Messerschmidt, 2005) and gender role strain (Pleck, 1995), demonstrating that Restrictive Emotionality, Dominance, and Toughness were significantly associated with aggression-related outcomes. Educational Attainment also emerged as a meaningful structural factor, whereas geographic setting did not show a significant effect. Together, these results underscore that male aggression is not simply a product of individual traits but reflects the interplay between psychological dispositions and socially reinforced gender expectations within specific structural contexts. To better understand the specific contributions of each predictor, follow-up univariate analyses were conducted, revealing targeted associations between individual masculinity norms and distinct forms of aggression.

### Masculinity norms and aggression

Across aggression domains, the masculinity norms Restrictive Emotionality, Dominance, and Toughness were the most consistent predictors of aggression-related outcomes.

In the aggression domain Mistrust, two masculinity dimensions, Restrictive Emotionality and Dominance, emerged as noteworthy predictors. Restrictive Emotionality reflects the suppression of vulnerability and avoidance of emotional openness. Men who strongly endorse this norm may be more inclined to perceive others as untrustworthy or threatening, consistent with research linking emotional restraint to impaired interpersonal trust and avoidant social behavior (Logoz et al., 2023). The internalization of such norms can lead to viewing social relationships as risky or unreliable, thereby reinforcing mistrust as a defensive adaptation. Dominance was also significantly associated with mistrust, suggesting that motives related to control and hierarchy contribute to distrustful social perceptions. Those who perceive relationships through a hierarchical or status-oriented lens may interpret others’ actions as competitive or undermining, fostering vigilance and suspicion, particularly in contexts where power and autonomy are central to masculine identity. This interpretation aligns with research showing that dominance-oriented masculinity is related to adversarial interpersonal schemas, reduced empathy, and a heightened tendency to interpret ambiguity as threat (Bosson et al., 2009; Weaver et al., 2010). Together, these findings indicate that emotional restriction and power orientation contribute independently to mistrust: the former through emotional withdrawal and the latter through defensiveness rooted in status concerns.

In the aggression domain Physical Trait Aggression, the masculinity dimensions Toughness and Dominance were the strongest predictors. Both capture core tenets of traditional masculinity, such as assertive control, physical strength, and emotional invulnerability, which are frequently reinforced in male peer culture, competitive environments, and institutional settings (Connell and Messerschmidt, 2005; Vandello and Bosson, 2013). Toughness reflects the idealization of emotional invulnerability and physical endurance, an expectation that men should endure pain, suppress vulnerability, and respond to threat with strength rather than reflection or withdrawal. Such ideals have been shown to limit emotional flexibility and promote risk-taking, competitiveness, and hostility when one’s strength or composure is challenged (Levant and Richmond, 2016; Hentschel et al., 2019). Within this framework, physical aggression may function both as a defense against perceived weakness and as a demonstration of conformity to cultural ideals of hardness and control.

Dominance, in contrast, represents the outward enactment of power through control or intimidation, complementing the inward focus on resilience reflected in Toughness. When combined, these dimensions create a potent configuration of masculine ideology: toughness legitimizes aggression as evidence of self-mastery, while dominance directs that aggression outward toward others in the assertion of hierarchy and authority. This combination helps explain why men endorsing both norms report the highest levels of physical trait aggression.

This is consistent with findings by Logoz et al. (2023), who showed that men endorsing traditional masculine norms, especially those tied to control and emotional hardness, report higher aggression and lower emotional competence. Their study suggests that emotional suppression and rigid status expectations may jointly contribute to aggressive tendencies, particularly in environments where dominance is validated. In the present study, the overall model predicting physical aggression yielded a large effect size (η^2^ = 0.368), indicating that a substantial portion of the variance was explained. However, the effect sizes of individual predictors were more modest (η^2^ ranging from 0.030 to 0.049), reflecting the combined influence of multiple masculinity dimensions and structural variables. These findings support the interpretation that aggression, particularly physical aggression, is shaped by a complex interplay of identity regulation, social pressure, and norm reinforcement. Men who endorse beliefs in male authority, hierarchical control over others, and an aversion to perceived weakness may use aggression not merely as an impulsive act, but as a socially sanctioned strategy for asserting dominance and maintaining status (Levant et al., 2011). This interpretation is consistent with Bosson et al. (2009) and Weaver et al. (2010). Their research shows that when men experience threats to their masculine identity, such as being disrespected or challenged, they often respond with aggression. This is especially true among men who strongly identify with dominance-oriented norms. In these contexts, aggression functions as a performative act that communicates power, deters threats, and reinforces alignment with dominant masculinity scripts. This is evident in male-on-male violence, school bullying, and street-level aggression, where toughness and control are currencies of respect (Messerschmidt, 2000; Anderson, 2008). These behaviors are reinforced by cultural narratives that link masculinity with physical prowess and emotional stoicism, portraying aggression as a legitimate or even necessary response to provocation.

The overall model for the aggression domain Verbal Trait Aggression displayed relatively weak associations overall, and no masculinity dimension remained significant after correction for multiple comparisons. This outcome suggests that verbal aggression may reflect more diffuse or situational dynamics not captured by the masculinity dimensions assessed here. Future research with larger and more diverse samples could further clarify whether antifemininity or other masculinity-related constructs meaningfully relate to verbal hostility.

Taken together, these findings affirm that masculinity norms are not only internalized but also actively performed and reinforced in social and institutional contexts. They also emphasize that interventions seeking to reduce aggression in men should not only address individual traits but should also target the institutional and cultural conditions that support rigid masculinity constructs.

### The structural role of education and geographic location

Educational attainment consistently predicted aggression outcomes, with lower levels of education associated with higher Mistrust and Physical Trait Aggression. These findings highlight education as a crucial structural context that shapes how masculinity norms are internalized and enacted. Educational settings can function as socializing environments that either reinforce or challenge hegemonic masculine ideals, depending on the extent to which they foster emotional literacy, empathy, and critical reflection on gender norms (Silva, 2022). Men with limited educational access may encounter fewer opportunities to question dominant gender ideologies or to develop alternative forms of self-expression and emotional regulation, thereby perpetuating narrower definitions of masculinity that normalize control and aggression.

The role of education extends beyond its association with socioeconomic status. It reflects differential exposure to cultural and psychological resources that can buffer against the pressures of traditional masculinity. Prior research indicates that men with lower educational attainment are more likely to endorse rigid gender norms and to engage in aggressive or coercive behavior (Ola, 2018). Conversely, higher education often involves exposure to more egalitarian values and social environments that promote cooperation and emotional openness. In this sense, education functions as an institutional context through which individuals encounter, negotiate, and potentially transform masculine ideals. It does so by shaping access to psychological resources, gender role alternatives, and normative models of behavior.

Understanding how educational structures intersect with gender ideology helps explain how social inequalities reinforce rigid masculinity norms and their behavioral consequences. These insights underscore the importance of educational settings as key contexts for preventive intervention and for promoting healthier forms of masculinity. Programs that incorporate emotional competence training, gender awareness, and critical reflection on masculinity ideals may help disrupt the reinforcement of aggressive norms and foster more adaptive, prosocial expressions of male identity. Further research should continue to examine how educational environments shape the relationship between masculinity and aggression, identifying the specific mechanisms through which learning contexts can facilitate attitudinal and behavioral change.

Although the present study did not find a significant effect of geographic setting, this absence may partly reflect the relatively homogeneous distribution of participants or the limited statistical power to detect small differences. Nonetheless, the findings suggest that educational experiences may exert a more immediate and measurable influence than geographic context. While spatial and cultural factors undoubtedly shape gender norms, education appears to provide a direct avenue for developing the cognitive and emotional competencies that mitigate aggression.

### Limitations and future directions

Several limitations should be acknowledged. First, the cross-sectional design precludes causal inference. Although the study identified significant associations between masculinity dimensions and aggression, it remains unclear whether adherence to traditional masculine norms promotes aggression or whether aggressive tendencies reinforce these norms. Longitudinal designs are needed to clarify the directionality and stability of these relationships over time.

Second, the nonsignificant effect of geographic location should be interpreted with caution. The uneven distribution of participants across categories likely reduced statistical power, limiting the ability to detect small but potentially meaningful contextual differences. While a *post hoc* sensitivity analysis indicated sufficient power for medium effects, smaller variations in gender-norm endorsement or aggression expression across urban and rural environments may have gone undetected.

Third, the sample consisted of men aged 18 to 40, primarily recruited through academic networks and social media. This may restrict the generalizability of the findings to men from other age groups or socioeconomic backgrounds, particularly those with limited internet access or differing educational opportunities. Moreover, other potentially influential structural variables, such as occupational background, cultural influences, and family context were not examined and may have contributed to the observed associations.

A limitation concerns the psychometric evaluation of the Swedish MRNI-SF. Although the instrument was translated and back-translated by bilingual psychologists to ensure conceptual and linguistic equivalence, a confirmatory factor analysis was not conducted within this study. To our knowledge, no Swedish adaptation of the MRNI-SF has undergone formal structural validation, such as confirmatory factor analysis, in previous research. The scale in the present study closely followed the established seven-factor structure of Levant et al. (2013) and demonstrated acceptable internal consistency (*α* = 0.70–0.83), supporting its use as a measure of traditional masculinity norms. However, the MRNI-SF primarily captures conventional masculinity ideals and does not encompass more inclusive or progressive forms of masculinity. Future research should employ broader instruments that assess diverse gender ideologies and adaptive expressions of masculinity.

Finally, although educational attainment and geographic setting were analyzed as structural factors, other socioeconomic and behavioral variables, such as income, occupational status, and alcohol use were not included and may confound the observed relationships. Future research should integrate these variables to more precisely delineate how structural and behavioral conditions intersect with masculinity and aggression.

Future studies should also adopt an explicitly intersectional approach to better capture the complexity of men’s social positions. In the Swedish context, class, migration background, and sexuality are particularly salient axes. Class and educational inequalities continue to influence access to social and emotional resources, while men from ethnic minority or immigrant backgrounds may face conflicting cultural expectations around masculinity. Moreover, heteronormative ideals persist despite progressive gender policies, constraining the expression of alternative masculinities. Examining these intersections would provide a more comprehensive understanding of how traditional masculinity operates within diverse social contexts and how it may contribute to or protect against aggression.

### Conclusion

This study contributes to the growing body of research linking traditional masculinity norms to aggression by demonstrating that specific masculine ideals, particularly those emphasizing dominance, toughness, and emotional restraint, are meaningfully associated with various forms of aggressive behavior. The results suggest that aggression functions not only as an individual disposition but also as a socially regulated and culturally reinforced expression of masculinity. Importantly, the findings reveal that different dimensions of masculinity are uniquely associated with distinct aggression outcomes. For instance, restrictive emotionality was closely linked to interpersonal mistrust, suggesting that emotional suppression may foster defensive or avoidant relational styles. In contrast, dominance and toughness were more strongly related to physical aggression, reflecting the role of power assertion and invulnerability in the enactment of masculinity through direct confrontation. Educational attainment consistently predicted aggression outcomes, with lower levels of education consistently associated with higher aggression. This pattern highlights how access to emotional literacy and opportunities for critical gender reflection may shape the internalization and expression of traditional masculinity norms. Programs that cultivate emotional competence, empathy, and critical reflection on gender norms have shown promise in reducing aggression (Levant and Richmond, 2016). Interventions should therefore focus not only on individual emotion regulation but also on transforming cultural and institutional norms that equate masculinity with dominance and control.

## Data Availability

The raw data supporting the conclusions of this article will be made available by the authors, without undue reservation.
